# Cellular localization of Y-box binding protein 1 in brain tissue of rats, macaques, and humans

**DOI:** 10.1186/1471-2202-10-28

**Published:** 2009-03-26

**Authors:** Bernadette Unkrüer, Anton Pekcec, Christina Fuest, Andrea Wehmeyer, Maria S Balda, Anja Horn, Wolfgang Baumgärtner, Heidrun Potschka

**Affiliations:** 1 Institute of Pharmacology, Toxicology, and Pharmacy, Ludwig-Maximilians-University Munich, Munich, Germany; 2 Devision of Cell Biology, Institute of Ophthalmology, University College London, London, UK; 3 Institute of Anatomy, Ludwig-Maximilians-University Munich, Munich, Germany; 4 Department of Pathology, University of Veterinary Medicine Hannover, Hannover, Germany

## Abstract

**Background:**

The Y-box binding protein 1 (YB-1) is considered to be one of the key regulators of transcription and translation. However, so far only limited knowledge exists regarding its cellular distribution in the adult brain.

**Results:**

Analysis of YB-1 immunolabelling as well as double-labelling with the neuronal marker NeuN in rat brain tissue revealed a predominant neuronal expression in the dentate gyrus, the cornu ammonis pyramidal cell layer, layer III of the piriform cortex as well as throughout all layers of the parahippocampal cortex. In the hilus of the hippocampus single neurons expressed YB-1. The neuronal expression pattern was comparable in the hippocampus and parahippocampal cortex of adult macaques and humans. Double-labelling of YB-1 with the endothelial cell marker Glut-1, the multidrug transporter P-glycoprotein, and the astrocytic marker GFAP did not indicate a co-localization. Following status epilepticus in rats, no induction of YB-1 occurred in brain capillary endothelial cells and neurons.

**Conclusion:**

In conclusion, our study demonstrates that YB-1 is predominantly expressed in neurons in the adult brain of rats, macaques and humans. Lack of a co-localization with Glut-1 and P-glycoprotein argues against a direct role of YB-1 in the regulation of blood-brain barrier P-glycoprotein.

## Background

Y-box binding proteins constitute a family of DNA/RNA-binding proteins that control gene expression at both the transcriptional and translational level [1,2]. The Y-box protein 1 (YB-1) represents a major mRNP (ribonucleoprotein particle) protein modulating the overall structure of mRNA to regulate translation [3]. With this function, YB-1 also represents one of the key regulators for expression of the multidrug transporter P-glycoprotein (Pgp) in tumor cells [4-10]. Pgp is known to confer cross-resistance to a variety of cytotoxic agents, thereby contributing to tumor resistance and therapeutic failure [11]. Huang et al. [12] demonstrated a direct link between YB-1 and Pgp expression in breast cancer cells and suggested YB-1 expression as a marker for chemoresistance, which may help to guide selection of the chemotherapy regime. Several studies confirmed that high YB-1 expression levels are associated with tumor aggressiveness, failure of chemotherapy, and a poor prognosis [13-16].

Over-expression of the multidrug transporter Pgp in blood-brain barrier (BBB) endothelial cells is discussed as one putative cause of drug-refractoriness in different CNS diseases such as epilepsy, depression, schizophrenia, neuroAIDS, and brain cancer [17]. Enhanced efflux function in brain capillary endothelial cells extrudes drugs into the capillary lumen, thereby reducing drug concentrations at brain parenchymal targets and contributing to therapeutic failure. Disease-associated factors as well as drugs are discussed to affect expression levels of Pgp in CNS diseases [17]. In view of the suggested role of Pgp in refractoriness, it is of specific interest to identify the factors which may contribute to regulation of Pgp in brain capillary endothelial cells. Based on data from tumor cells, we hypothesized that YB-1 may also co-localize with Pgp in brain capillary endothelial cells.

Besides induction of Pgp expression, numerous further changes occur in gene expression in a variety of cell types following seizure activity in the epileptic brain as well as during the development of symptomatic epilepsy [18,19]. Whereas some of these changes just reflect consequences of the insult others may critically contribute to the formation, stabilization, or extension of a hyperexcitable network. In this context knowledge about the regulators involved in the complex transcriptome and proteome changes would increase the understanding of the mechanisms of disease progression and epileptogenesis.

Therefore, it is of specific interest to further elucidate the role of the transcriptional and translational regulator YB-1 in the brain. In the present study, we investigated the cellular localization of YB-1 in brain tissue of rats, macaques and humans. In view of a putative role of YB-1 in the regulation of Pgp in brain capillaries, we tested if YB-1 co-localizes with Pgp in brain capillary endothelial cells. In order to gain knowledge about an impact of YB-1 in the pathophysiology of the diseased brain, the expression of YB-1 was determined in the early phase following a status epilepticus.

## Methods

### Animals and Tissue Information

Female Wistar Unilever rats were purchased at a body weight of 180–200 g (Harlan-Winkelmann, Borchen, Germany). Rats were kept under controlled environmental conditions (24–25°C; humidity 50–60%; 12 hour dark/light cycle) with free access to tap water and food. Before being used in the experiments, the rats were allowed to adapt to the new conditions for at least 1 week. All experiments were done in compliance with the European Communities Council Directive of 24 November 1986 (86/609/EEC). All efforts were made to minimize pain or discomfort of the animals used.

YB-1 expression was studied in the brain of old world primates and humans. Therefore, brain tissue from a six year old adult male macaque (Macaca fascicularis), that had been transcardially perfused with 4% paraformaldehyde in 0.1 M phosphate buffer was obtained and processed as described in detail elsewhere [20]. Brain blocks containing either the hippocampus and parahippocampal cortex or the parietal cortex were cut in 40 μm sections using a cryostat (HM 560; Microm, Walldorf, Germany) and were stored at -20°C in cryoprotecting solution (glycerol and 0.1 M phosphate buffer, pH 7.4, 1:1 in volume). In addition, 3 μm thin paraffin sections of hippocampus from a former neuropathological study (for detail see [21]) were used to evaluate YB-1 expression in the human brain. The brain tissue of a forty-four year old male subject that lacked any neuropathological alterations served as a control in our study.

### Induction of Status Epilepticus in rats

For induction of status epilepticus in rats (n = 40) lithium chloride (127 mg/kg i.p., Sigma, Taufkirchen, Germany) was administered 14 h and methyl-scopolamine (1 mg/kg i.p., Sigma; Taufkirchen, Germany) was administered 30 min before the first pilocarpine injection. As described previously [22] pilocarpine (Sigma, Taufkirchen, Germany) was given intraperitoneally (10 mg/kg) every 30 min until the onset of convulsive status epilepticus (SE) consisting of ongoing generalized convulsive seizures. The total number of pilocarpine injections was limited to 12 per animal. Seizure activity was monitored behaviourally. Status epilepticus was terminated after 90 min by injection of diazepam (10 mg/kg). If seizure activity continued, diazepam administration was repeated after 3 minutes. Only rats exhibiting continuous convulsive seizure activity during status epilepticus were used for further analysis. Control rats (n = 8) were treated similarly, but saline was given instead of pilocarpine and methyl-scopolamine.

### Tissue Preparation

For studying YB-1 expression vehicle treated control rats (n = 8) and rats with intervals of 2 h (n = 2), 4 h (n = 6), 8 h (n = 7), and 48 h (n = 6) following status epilepticus were decapitated. The brains were immediately removed, embedded in Tissue Freezing Medium^® ^(Jung, Nussloch, Germany), frozen in liquid nitrogen and stored at -80°C. The tissue was cut at 14 μm using a cryostat (HM 560; Microm, Walldorf, Germany) and sections were mounted onto HistoBond^® ^adhesion slides (Marienfeld, Lauda-Koenigshofen, Germany). For YB-1/NeuN double-labelling additional naïve rats were deeply anesthetized with chloralhydrate and were transcardially perfused with saline followed by 4% paraformaldehyde in 0.1 M phosphate buffered saline (pH 7.4). The brains were removed and transferred into 30% sucrose and stored at 4°C until cutting at 40 μm on a cryostat (HM 560; Microm, Walldorf, Germany) in the coronal plane. Sections were stored at -20°C in cryoprotecting solution (glycerol and 0.1 M phosphate buffer, pH 7.4, 1:1 in volume).

### YB-1 immunostaining

For YB-1 immunohistochemistry frozen sections of rats were incubated in acetone for 10 minutes at -20°C. Sections were allowed to air dry at room temperature for two days, rinsed in 0.05 M Tris-buffered saline (TBS; pH 7.6) and incubated in 0.5% TBS buffered H_2_O_2 _for 30 minutes. Following a pre-incubation in a blocking solution containing 2% bovine serum albumin, 0.3% Triton X-100, and 5% normal goat serum (serum was chosen depending on host species of the secondary antibody) in TBS for 60 minutes, sections were incubated in primary antiserum containing polyclonal rabbit anti-YB-1 (Abcam, Cambridge, UK) overnight at 4°C. Then, sections were rinsed in TBS, placed in biotin-labelled goat anti-rabbit (Jackson Immunoresearch Laboratories, West Grove, PA, USA), 1:200 for 90 minutes, and after rinsing again in TBS sections were incubated in horseradish peroxidase-labelled streptavidin (DAKO, Hamburg, Germany), 1:375 for 90 minutes. After several TBS rinses the applied antibodies were visualized by a nickel-intensified diaminobenzidine [19] reaction (0.05% 3,3-diaminobenzidine, 0.01%, nickel ammonium sulphate; both from Sigma, Taufkirchen, Germany, and 0.01% H_2_O_2_). After washing, the sections were mounted onto glass slides, air dried, dehydrated, and coverslipped with Entellan (Merck, Darmstadt, Germany).

For YB-1 immunohistochemistry of the macaque brain tissue, the same protocol was applied as described for rats except that the sections were processed free-floating without acetone and drying pre-treatment.

Formalin-fixed, paraffin-embedded brain tissue was first deparaffinised, rehydrated and pre-treated as described previously [21,23]. Then, sections were processed as described for rat and macaque tissue.

For the combined detection of YB-1/Glut-1, sections of rat, macaque and human brain tissue were preincubated in donkey serum (Jackson Immunoresearch Laboratories, West Grove, PA, USA). Then the sections were incubated in a mixture of polyclonal rabbit anti-YB-1 (Abcam, Cambridge, UK), 1:250 and monoclonal mouse anti-Glut-1 antibody (Abcam, Cambridge, UK), 1:500. After rinsing, the sections were reacted with a carbocyanin 3-labelled donkey anti-rabbit (Jackson Immunoresearch Laboratories, West Grove, PA, USA), 1:500, and biotinylated donkey anti-mouse, 1:500, in combination with carbocyanin 2-conjugated streptavidin, 1:500 (both from Jackson Immunoresearch Laboratories, West Grove, PA, USA) for 90 min at room temperature.

The simultaneous immunodetection of YB-1 and either the neuron-specific nuclear antigen (NeuN) or glial fibrillary acid protein (GFAP) was performed on free-floating 40 μm thick sections of paraformaldehyde-perfused rats and macaques. The sections were treated with a mixture of polyclonal rabbit anti-YB-1 antibody (Abcam Cambridge, UK) and either monoclonal biotinylated mouse anti-NeuN antibody or monoclonal mouse anti-GFAP antibody (both from Millipore former Chemicon, Hofheim, Germany) was used. The respective secondary antibodies anti-rabbit and anti-mouse IgG (Jackson Immunoresearch Laboratories, West Grove, PA, USA) were used at a dilution of 1:500.

For combined immunodetection of YB-1 and Pgp frozen rat brain sections were pretreated as described above for YB-1 single-labelling, but preincubated in donkey serum instead of goat serum. Then, sections were incubated in a mixture of polyclonal rabbit anti-YB-1 (Abcam Cambridge, UK), 1:250 and polyclonal goat anti-Pgp antibody (Santa Cruz Biotechnology, Heidelberg, Germany), 1:30, overnight at 4°C. Carbocyanin 3-labelled donkey anti-rabbit antibody, 1:500, as well as biotinylated donkey anti-goat antibody, 1:500, in combination with carbocyanin 2-conjugated streptavidin, 1:500 (all from Jackson Immunoresearch Laboratories, West Grove, PA, USA) were used as secondary antibodies.

### Immunohistological evaluation and Statistics

The DAB-stained sections was examined with a Olympus BH2 microscope with Plan-Neofluar lenses (Zeiss, Göttingen, Germany) equipped with a single chip charge-coupled device (CCD) color camera (Axiocam; Zeiss, Göttingen, Germany), and connected with an AMD Athlon^64 ^Processor-based computer with an image capture interface card (Axiocam MR Interface Rev.A; Zeiss, Göttingen, Germany). The immunolabelling was analyzed in different brain regions that are known to be involved in epileptic circuits, i.e. subregions of the hippocampus, in the parahippocampal cortex, and in rat tissue also in the piriform cortex. Available samples from the macaque and human brain only included the hippocampus and parahippocampal tissue.

For counting of YB-1 positive hilar neurons, the signal of the immunoperoxidase-treated sections was captured with the computer-assisted imaging system StereoInvestigator 6.0 (Microbrightfield Europe, Magdeburg, Germany). The hardware consists of a Leica DMLB microscope (Leica, Bensheim, Germany), a Plan-Neofluar lens (Leica, Bensheim, Germany), a single chip charge coupled device (CCD) color camera (CX9000, Microbrightfield Europe, Magdeburg, Germany), and an AMD Athlon (tm) 64 Processor. An experimenter blinded to the treatment conditions traced the extent of the hippocampal dentate hilus and performed the counting of cells using the optical fractionator method. In slide-mounted sections the area of the dentate hilus was traced and within each traced contour a step grid was placed. Counting frames were automatically and randomly placed along the grid. The thickness of the counting frame was equal to the thickness of the section (minus guard zones from the top and bottom of the section). Only immunoreactive cells which appeared within the counting frame and came into focus were counted.

Fluorescent signals from double-labelled sections were analyzed using a confocal microscope (Leica TCS SP2; Leica, Bensheim, Germany). For evaluation of double-labelling or lack of double-labelling confocal z-series of cells were carefully analyzed.

Statistical differences in cellular counts were analyzed by one way analysis of variance followed by the Student's t-test (unpaired). Data are expressed as means ± SEM. A p < 0.05 was considered significant and is indicated by an asterisk.

## Results

### YB-1 expression in the rat, macaque, and human brain

**Figure 1:**
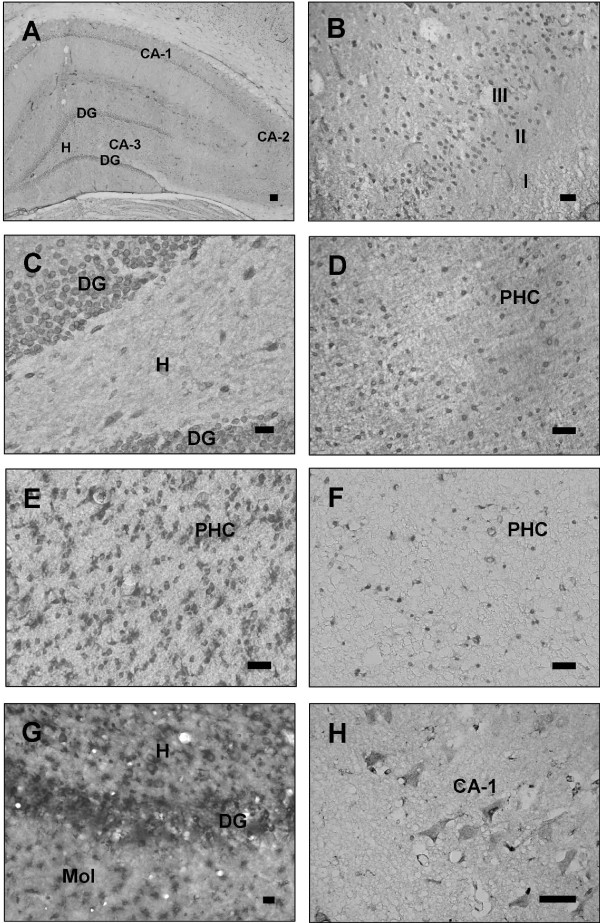
**Micrographs showing expression of the transcription factor YB-1 in the rat, macaque and human brain (A-H)**. (A) Image displaying hippocampal YB-1 expression visualized by a DAB-staining method in a 14 μm thin section of an adult rat's hippocampus (DG = dentate gyrus, H = hilus of the dentate gyrus, CA = cornu ammonis formation). (B) Representative example of YB-1 expression in the piriform cortex (layer I-III) of an adult rat. Note, that the expression of YB-1 is predominantly related to cells in the layer III of the piriform cortex. (C) Image showing YB-1 expressing granule cells of the dentate gyrus and YB-1 expressing cells in the hilar (H) subregion of the hippocampus of an adult rat. (D) Image displaying YB-1 expression visualized by a DAB-staining method in a 14 μm thick section of the parahippocampal cortex (PHC) of a rat. YB-1 expression appears throughout all layers of the cortex. (E) Image displaying YB-1 expression visualized by a DAB-staining method in a 40 μm thick section of a macaque's parahippocampal cortex (PHC). In this image YB-1 appears to be stronger expressed as in the rat's brain due to the relative thickness of this section in comparison to the thinner sections of the rat. (F) Image displaying YB-1 expression visualized by a DAB-staining method in a 3 μm thin section of a human parahippocampal cortex (PHC). Due to the section thickness of only 3 μm, YB-1 appears to be less expressed compared to images of the rat's and macaque's brain. (G) Image displaying YB-1 expressing granule cells of the dentate gyrus (DG) as well as YB-1 expressing cells in the hilar (H) subregion and the molecular layer (Mol) of the hippocampal formation in the brain of a macaque. (H) YB-1 expression in the hippocampal CA-1 region of the human brain. A: scale bar = 100 μm. B-H: scale bar = 20 μm.

**Figure 2:**
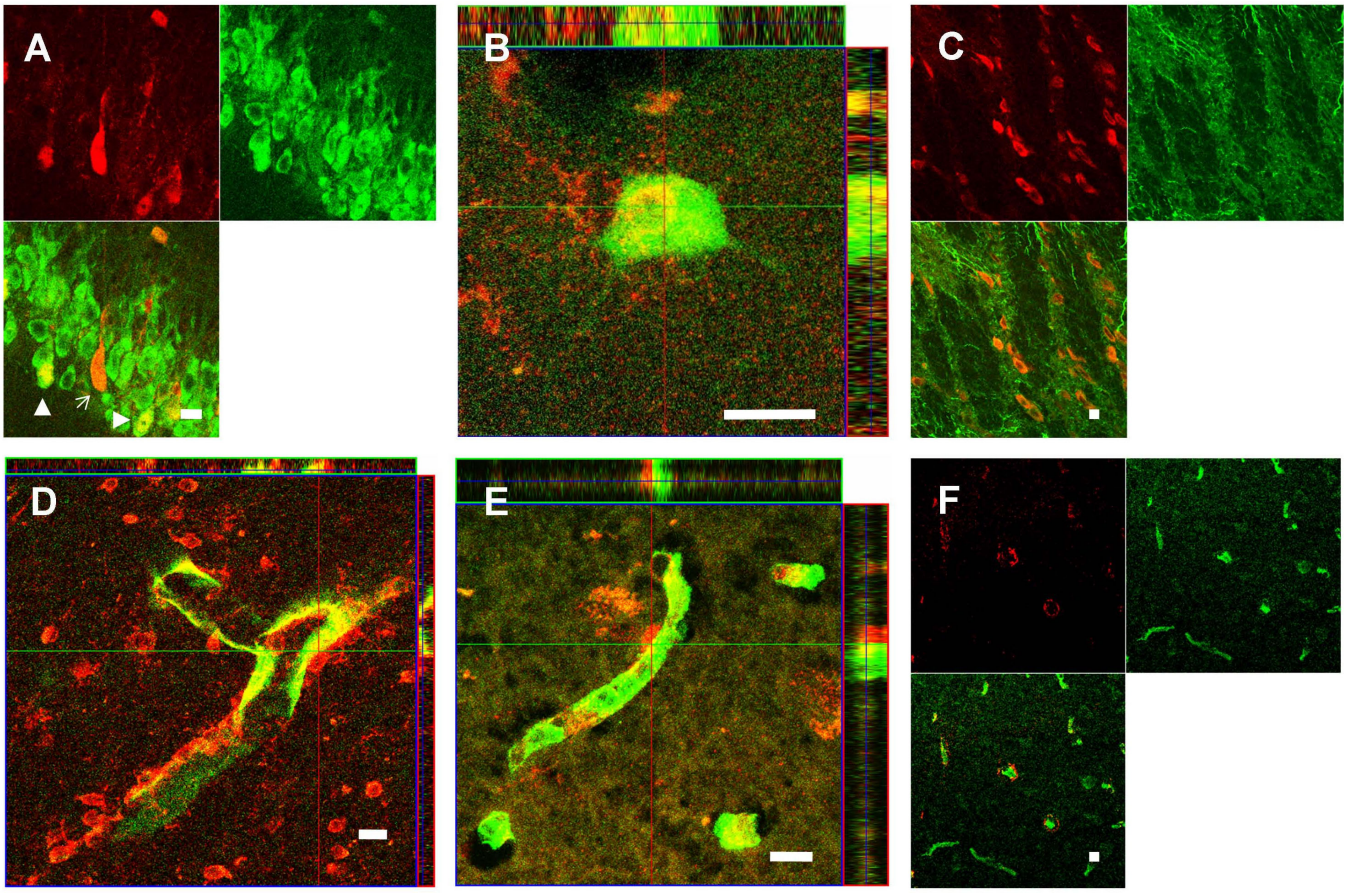
**Micrographs showing multiple-labelled tissue of the rat, macaque and human brain (A-F)**. (A) Split image displaying labelling of YB-1 (red) and NeuN (green) and double-labelling (merged figure) in the hippocampal cornu ammonis (CA-1) formation of a rat brain. Representative examples of cells showing nuclear YB-1 expression are indicated by bold white arrowheads while a narrow arrow indicates a cell with cytoplasmatic expression of YB-1. (B) High magnification image of a neuron indicated by NeuN expression (green) with a clear nuclear staining of YB-1 (red) in the parietal cortex of a macaque. (C) Split image showing the expression of YB-1 (red) and the glial marker GFAP (green) in the hilus of the dentate gyrus of a rat. Note the absence of any glial expression of YB-1 in the merged figure. (D, E) Confocal image showing the expression of YB-1 (red) and the expression of the endothelial cell marker Glut-1 (green) in the macaque and human brain. (D) Image displaying the expression of YB-1 (red) and Glut-1 (green) in the parahippocampal cortex of a macaque monkey. Note the lack of co-localization of YB-1 and Glut-1 expression, as clearly indicated by confocal z-series. (E) Image displaying the expression of YB-1 (red) and Glut-1 (green) in the human parahippocampal cortex. In accordance with data obtained from rats (data not shown) and macaques, YB-1 proved not to be expressed by Glut-1 positive endothelial cells. (F) Split image of a confocal micrograph giving an example of YB-1 expression (red) and expression of the endothelial multidrug transporter Pgp (green) in the parietal cortex of a rat. In accordance with the data from the YB-1/Glut-1 double-labelling in the different species, YB-1 does not co-localize with endothelial Pgp expression (merged figure). Scale bar = 10 μm.

Analysis of YB-1 immunolabelling in rat brain tissue revealed a strong expression in the dentate gyrus, the cornu ammonis pyramidal cell layer, layer III of the piriform cortex, as well as throughout all layers of the parahippocampal cortex (Fig. 1A–D). Based on the morphology the vast majority of labelled cells presented a neuronal phenotype. An identical expression pattern was observed in the hippocampus and the parahippocampal cortex of macaques and humans (Fig. 1E–H). In the hilus of the hippocampus of all three species single YB-1 positive neurons were evident. Expression of YB-1 in neurons was substantiated by double-labelling with the neuronal cell marker NeuN. In rat and macaque tissue representative YB-1 immunoreactive cells with a neuronal morphology were carefully analyzed on serial optical planes. This analysis confirmed a co-localization of YB-1 and NeuN (Fig. 2A–B). The majority of cells showed a dominant YB-1 expression in the cytoplasm, whereas a predominant nuclear localization of YB-1 was obvious in some cells only (Fig. 2A–B). In contrast, there was no evidence for a co-localization of YB-1 with the astroglial cell marker GFAP in different regions of the rat brain (Fig. 2C). Thereby these data imply that YB-1 is not expressed in astrocytes in the naïve brain to a relevant extent.

Furthermore the microscopic analysis of the tissue of all three species indicated an expression of YB-1 in association with brain vessels (Fig. 1A).

### Lack of co-localization with the endothelial cell marker Glut-1 and with Pgp

In brain tissue of rats, macaques, and humans microvessels in all brain regions were intensely stained by Glut-1 immunolabelling. The careful analysis of double Glut-1/YB-1 immunolabelled sections in serial optical planes did not reveal any evidence for a YB-1 expression in Glut-1 immunolabelled endothelial cells (Fig. 2D, E).

In all regions of the rat brain tissue, microvessels were heavily stained by Pgp immunolabelling. On longitudinal as well as cross-sectioned microvessels, Pgp labelling outlined the entire vessel profile, and corresponded to the endothelial cells at the vessel walls (Fig. 2F). With the immunohistological procedure used in the present study, Pgp-specific staining was not observed in neurons or glial cells. The analysis of serial optical planes of double Pgp/YB-1 immunolabelled sections did not indicate co-localization of YB-1 with endothelial Pgp (Fig. 2F).

### YB-1 expression following status epilepticus

Seventy-five percent of the rats (n = 30 out of 40) developed a status epilepticus with generalized convulsive seizures in response to repeated injections of the cholinomimetic pilocarpine. The rats required a mean dosage of 32.7 ± 3.59 mg/kg pilocarpine (mean ± SEM) to develop a status epilepticus. All rats exhibited a comparable ongoing generalized seizure activity continuing until administration of diazepam.

In brain capillary endothelial cells YB-1 expression was not induced by status epilepticus. Analysis of Glut-1/YB-1 or Pgp/YB-1 double-labelled sections of representative samples did not reveal an expression of YB-1 in endothelial cells at different time points following prolonged seizure activity.

**Figure 3:**
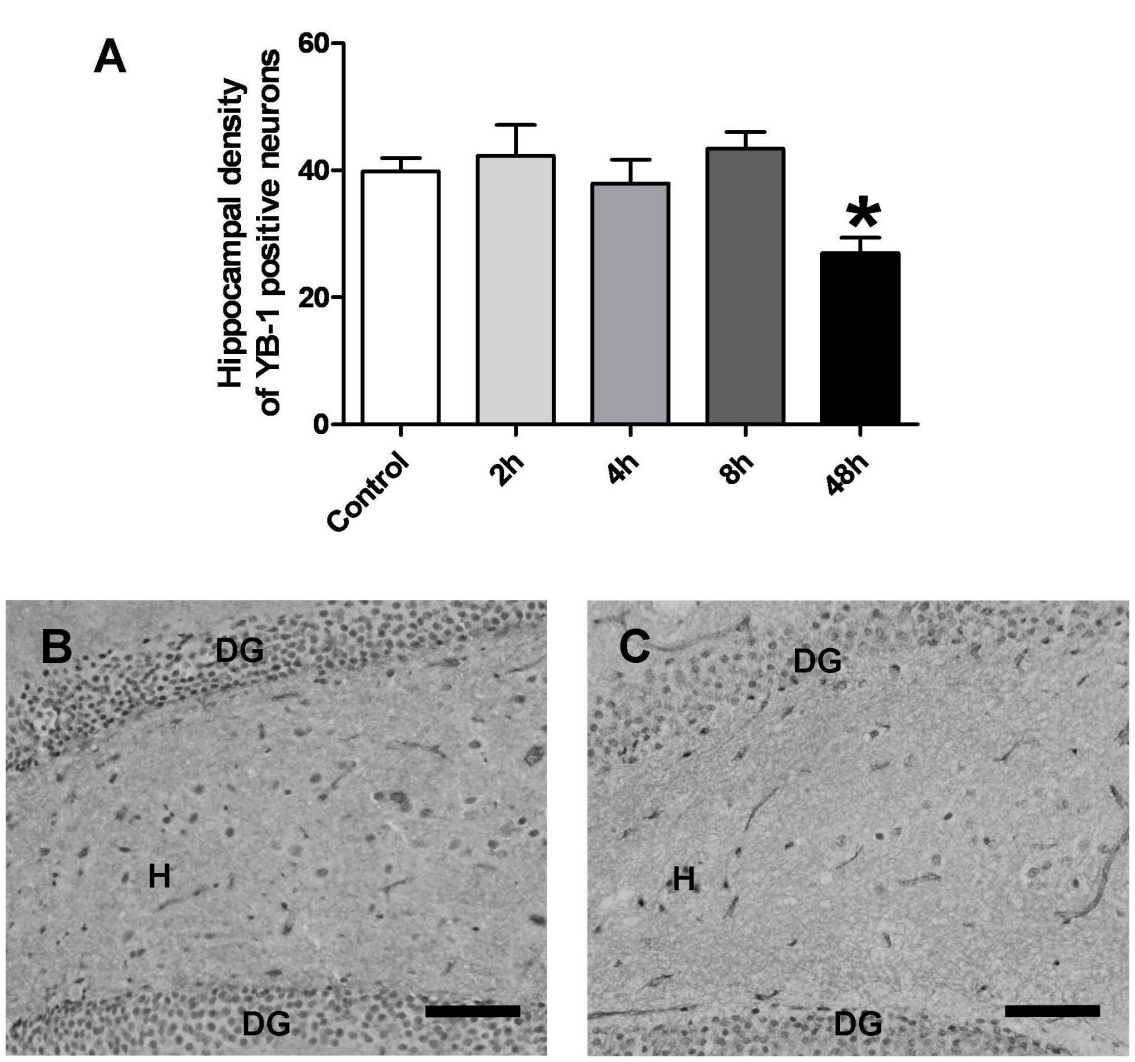
**Analysis of neural YB-1 expression in the hippocampal hilus**. Data are given as neural density (mean ± SEM). Significant differences to controls are indicated by an asterisk. One way analysis of variance indicated that groups differ significantly (p = 0.0044). (A) Analysis of the hilar density of YB-1 reactive neurons two (n = 2), four (n = 6) or eight hours (n = 7) after status epilepticus did not show significant differences to controls (n = 8) (in all cases p > 0.05). In contrast, the neural density of YB-1 expressing cells was decreased 48 hours (n = 6) after status epilepticus (p = 0.0017). (B) Representative image of the dentate hilus (H) of a control rat. (C) Representative image of the dentate hilus (H) of a rat 48 hours after status epilepticus. Note the tremendous decrease in YB-1 expressing hilar neurons. (DG = dentate gyrus). Scale bar = 100 μm.

The overall expression pattern of YB-1 in hippocampal cells with a neuronal morphology did not change during the first 48 h following SE. No induction was observed in hippocampal cells at the different time points investigated (Fig. 3). The significant reduction 48 h following SE is likely to be related to hippocampal cell loss that is known to be evident at this time point (Fig. 3).

## Discussion

YB-1 is a DNA- and RNA-binding protein that functions as a sequence specific transcriptional and translational regulator. Here, we demonstrated that in the adult brain of rats, macaques, and humans YB-1 is predominantly expressed in neurons. So far postnatal expression in neurons has only been described in mouse and rat brain based on Western Blot analysis of brain tissue extracts or on studies using primary cultures of hippocampal neurons [24,25]. Funakoshi et al. [25] described a decline of mouse and rat brain expression of YB-1 in the postnatal phase. Miwa and colleagues [24] reported a hippocampal expression that was prominent at the age of 5 days but decreased significantly in 4-week old mice. However, neuronal expression was not confirmed by immunohistological double-labelling in these studies and no data were given about the hippocampal subregions. Our analysis revealed a prominent expression in granule cells of the dentate gyrus as well as an expression in the cornu ammonis pyramidal cell layer. Only single cells were labelled in the hilus of the hippocampus. In comparison to rat and macaque tissue labelling was less pronounced in human tissue. This might be related to the processing of the tissue or to differences in the affinity of the antibody to the YB-1 isoforms of the different species. In the parahippocampal cortex of all species YB-1 neuronal expression was evident with a distribution that appeared to be independent of the lamination of the cortex. In adult rat brain tissue an additional analysis of YB-1 in the piriform cortex revealed an association with layer III. Neuronal YB-1 expression has previously been reported in the developing brain of mice as well as of a human 24-week old foetus [26-28]. Our data indicate that neuronal YB-1 expression in specific brain regions extends to the adult brain and reaches relevant levels in the brain of adult rats, macaques, and humans. Predominant localization in the cytoplasm indicates that a large sub-fraction of YB-1 exists in a functional standby state. These data are in accordance with previous reports. Studies in primary cultures of hippocampal neurons as well as studies in early postnatal mouse brain tissue have also indicated a primary cytoplasmic localization [24,25]. Nuclear YB-1 represents the sub-fraction available for interference with DNA and RNA which is in the appropriate localization to regulate the transcriptome and proteome of the cells. Nuclear translocation proved to act as a regulatory mechanism in response to a variety of stresses such as hyperthermia-associated cell stress in human colon carcinoma cells [29] and treatment with the cytostatic agent paclitaxel in breast cancer patients [30].

YB-1 has been reported to represent one of the key regulators for expression of the multidrug transporter Pgp in tumor cells [4-10]. Expression levels of the efflux transporter Pgp are of specific interest as Pgp confers cross-resistance to a variety of cytotoxic agents [11]. Enhanced efflux of cytostatic drugs decreases concentrations at the target sites thereby contributing to tumor resistance and therapeutic failure. Several studies confirmed that high YB-1 expression levels are linked with tumor aggressiveness, failure of chemotherapy, and a poor prognosis in different tumor types [12-16]. BBB Pgp is discussed as a contributing factor in drug-refractoriness of various CNS diseases including epilepsy, brain ischemia, HIV encephalopathy, and psychiatric diseases [17]. Interestingly, evidence exists that Pgp expression can be driven by pathophysiological mechanisms or by drug therapy [17]. Recently, we reported that a glutamate/NMDA receptor/cyclooxygenase-2 pathway is involved in the induction of Pgp in the epileptic brain [22]. However, the downstream effectors of the cascade still need to be identified. Furthermore translational regulation may also affect expression levels in the diseased brain. Based on the link between YB-1 and tumor Pgp, we hypothesized that YB-1 may also regulate Pgp expression in brain capillary endothelial cells. However, double-labelling studies with the endothelial cell marker Glut-1 and with Pgp did not confirm an expression of YB-1 in endothelial cells. Moreover, no induction of YB-1 was observed in brain endothelial cells following prolonged seizure activity in a rat status epilepticus model. Therefore, YB-1 is apparently not involved in transcriptional and translational regulation in brain capillary endothelial cells neither in the healthy brain nor in the epileptic brain. Lack of co-localization of YB-1 and the astrocytic marker GFAP indicates that this conclusion applies to astrocytes as well. As the majority of astrocytic endfeet are labelled for GFAP, we can rule out that the YB-1 expression observed in association with brain microvessels is due to an astrocytic expression. The question remains to be open, whether YB-1 expression in the vicinity of the vascular unit might be related to pericytes. Detection in pericytes would indicate a role of YB-1 in transcriptional and translational regulation in these cells which might affect the overall expression pattern and activity status. As the role of pericytes was not in the scope of our interest, we did not further analyze double-labelling with pericyte markers.

YB-1 expression was not affected in hippocampal neurons in the early hours following status epilepticus. This result argues against a key role of the transcriptional and translational regulator YB-1 for the complex changes in the transcriptome and proteome of the hippocampus that occur following seizure activity [18,19]. However, it needs to be considered that the functional state of YB-1 might be additionally affected in all brain regions by changes in its nuclear translocation, which was not assessed in the status epilepticus model in the present study. The reduction in YB-1 labelled cells 48 hours is likely to be associated with hippocampal cell loss, which is known to be evident at this time point.

## Conclusion

In conclusion, the present study demonstrated neuronal expression of YB-1 in the hippocampus and parahippocampal cortex of adult rats, macaques, and humans. In addition, analysis of rat brain tissue revealed an expression in the piriform cortex. Double-labelling studies indicated that YB-1 is not expressed in brain capillary endothelial cells and in astrocytes. Moreover, a co-localization with Pgp was ruled out arguing against a role of YB-1 in the regulation of blood-brain barrier Pgp expression.

## Authors' contributions

BU and AP performed all the experiments reported in this manuscript, developed the methods, contributed to the study design and drafting of the manuscript. AW performed the stereological cell counting and contributed, together with CF, to the figure generation. MSB contributed to the concept of the study. AH and WB provided brain tissue of macaque and human brain, respectively. HP is the senior author, in whose laboratory this work was performed, who conceived the concept and the design of the study, and who drafted the manuscript. All authors read and approved the final manuscript.

## References

[B1] Kohno K, Izumi H, Uchiumi T, Ashizuka M, Kuwano M (2003). The pleiotropic functions of the Y-box-binding protein, YB-1. Bioessays.

[B2] Evdokimova V, Ovchinnikov LP, Sorensen PH (2006). Y-box binding protein 1: providing a new angle on translational regulation. Cell Cycle.

[B3] Skabkin MA, Kiselyova OI, Chernov KG, Sorokin AV, Dubrovin EV, Yaminsky IV, Vasiliev VD, Ovchinnikov LP (2004). Structural organization of mRNA complexes with major core mRNP protein YB-1. Nucleic Acids Res.

[B4] Bargou RC, Jurchott K, Wagener C, Bergmann S, Metzner S, Bommert K, Mapara MY, Winzer KJ, Dietel M, Dorken B (1997). Nuclear localization and increased levels of transcription factor YB-1 in primary human breast cancers are associated with intrinsic MDR1 gene expression. Nat Med.

[B5] Oda Y, Sakamoto A, Shinohara N, Ohga T, Uchiumi T, Kohno K, Tsuneyoshi M, Kuwano M, Iwamoto Y (1998). Nuclear expression of YB-1 protein correlates with P-glycoprotein expression in human osteosarcoma. Clin Cancer Res.

[B6] Ohga T, Uchiumi T, Makino Y, Koike K, Wada M, Kuwano M, Kohno K (1998). Direct involvement of the Y-box binding protein YB-1 in genotoxic stress-induced activation of the human multidrug resistance 1 gene. J Biol Chem.

[B7] Kamura T, Yahata H, Amada S, Ogawa S, Sonoda T, Kobayashi H, Mitsumoto M, Kohno K, Kuwano M, Nakano H (1999). Is nuclear expression of Y box-binding protein-1 a new prognostic factor in ovarian serous adenocarcinoma?. Cancer.

[B8] Oda Y, Ohishi Y, Saito T, Hinoshita E, Uchiumi T, Kinukawa N, Iwamoto Y, Kohno K, Kuwano M, Tsuneyoshi M (2003). Nuclear expression of Y-box-binding protein-1 correlates with P-glycoprotein and topoisomerase II alpha expression, and with poor prognosis in synovial sarcoma. J Pathol.

[B9] Saji H, Toi M, Saji S, Koike M, Kohno K, Kuwano M (2003). Nuclear expression of YB-1 protein correlates with P-glycoprotein expression in human breast carcinoma. Cancer Lett.

[B10] Kuwano M, Uchiumi T, Hayakawa H, Ono M, Wada M, Izumi H, Kohno K (2003). The basic and clinical implications of ABC transporters, Y-box-binding protein-1 (YB-1) and angiogenesis-related factors in human malignancies. Cancer Sci.

[B11] Borst P, Jonkers J, Rottenberg S (2007). What makes tumors multidrug resistant?. Cell Cycle.

[B12] Huang J, Tan PH, Li KB, Matsumoto K, Tsujimoto M, Bay BH (2005). Y-box binding protein, YB-1, as a marker of tumor aggressiveness and response to adjuvant chemotherapy in breast cancer. Int J Oncol.

[B13] Janz M, Harbeck N, Dettmar P, Berger U, Schmidt A, Jurchott K, Schmitt M, Royer HD (2002). Y-box factor YB-1 predicts drug resistance and patient outcome in breast cancer independent of clinically relevant tumor biologic factors HER2, uPA and PAI-1. Int J Cancer.

[B14] Gimenez-Bonafe P, Fedoruk MN, Whitmore TG, Akbari M, Ralph JL, Ettinger S, Gleave ME, Nelson CC (2004). YB-1 is upregulated during prostate cancer tumor progression and increases P-glycoprotein activity. Prostate.

[B15] Vaiman AV, Stromskaya TP, Rybalkina EY, Sorokin AV, Ovchinnikov LP, Stavrovskaya AA (2007). Development of drug resistance in the population of colon cancer cells under the effect of multifunctional protein YB-1. Bull Exp Biol Med.

[B16] Oda Y, Ohishi Y, Basaki Y, Kobayashi H, Hirakawa T, Wake N, Ono M, Nishio K, Kuwano M, Tsuneyoshi M (2007). Prognostic implications of the nuclear localization of Y-box-binding protein-1 and CXCR4 expression in ovarian cancer: their correlation with activated Akt, LRP/MVP and P-glycoprotein expression. Cancer Sci.

[B17] Loscher W, Potschka H (2005). Drug resistance in brain diseases and the role of drug efflux transporters. Nat Rev Neurosci.

[B18] Aronica E, Gorter JA (2007). Gene expression profile in temporal lobe epilepsy. Neuroscientist.

[B19] Lukasiuk K, Dabrowski M, Adach A, Pitkanen A (2006). Epileptogenesis-related genes revisited. Prog Brain Res.

[B20] Horn AK, Eberhorn A, Hartig W, Ardeleanu P, Messoudi A, Buttner-Ennever JA (2008). Perioculomotor cell groups in monkey and man defined by their histochemical and functional properties: reappraisal of the Edinger-Westphal nucleus. J Comp Neurol.

[B21] Czasch S, Paul S, Baumgartner W (2006). A comparison of immunohistochemical and silver staining methods for the detection of diffuse plaques in the aged canine brain. Neurobiol Aging.

[B22] Bauer B, Hartz AM, Pekcec A, Toellner K, Miller DS, Potschka H (2008). Seizure-induced up-regulation of P-glycoprotein at the blood-brain barrier through glutamate and cyclooxygenase-2 signaling. Mol Pharmacol.

[B23] Pekcec A, Baumgartner W, Bankstahl JP, Stein VM, Potschka H (2008). Effect of aging on neurogenesis in the canine brain. Aging Cell.

[B24] Miwa A, Higuchi T, Kobayashi S (2006). Expression and polysome association of YB-1 in various tissues at different stages in the lifespan of mice. Biochim Biophys Acta.

[B25] Funakoshi T, Kobayashi S, Ohashi S, Sato TA, Anzai K (2003). Isolation and characterization of brain Y-box protein: developmentally regulated expression, polyribosomal association and dendritic localization. Brain Res Mol Brain Res.

[B26] Spitkovsky DD, Royer-Pokora B, Delius H, Kisseljov F, Jenkins NA, Gilbert DJ, Copeland NG, Royer HD (1992). Tissue restricted expression and chromosomal localization of the YB-1 gene encoding a 42 kD nuclear CCAAT binding protein. Nucleic Acids Res.

[B27] Ohba H, Chiyoda T, Endo E, Yano M, Hayakawa Y, Sakaguchi M, Darnell RB, Okano HJ, Okano H (2004). Sox21 is a repressor of neuronal differentiation and is antagonized by YB-1. Neurosci Lett.

[B28] Liverman CS, Kaftan HA, Cui L, Hersperger SG, Taboada E, Klein RM, Berman NE (2006). Altered expression of pro-inflammatory and developmental genes in the fetal brain in a mouse model of maternal infection. Neurosci Lett.

[B29] Stein U, Jurchott K, Walther W, Bergmann S, Schlag PM, Royer HD (2001). Hyperthermia-induced nuclear translocation of transcription factor YB-1 leads to enhanced expression of multidrug resistance-related ABC transporters. J Biol Chem.

[B30] Fujita T, Ito K, Izumi H, Kimura M, Sano M, Nakagomi H, Maeno K, Hama Y, Shingu K, Tsuchiya S (2005). Increased nuclear localization of transcription factor Y-box binding protein 1 accompanied by up-regulation of P-glycoprotein in breast cancer pretreated with paclitaxel. Clin Cancer Res.

[B31] Brem H, Ewend MG, Piantadosi S, Greenhoot J, Burger PC, Sisti M (1995). The safety of interstitial chemotherapy with BCNU-loaded polymer followed by radiation therapy in the treatment of newly diagnosed malignant gliomas: phase I trial. J Neurooncol.

